# The effect of citicoline administration on visual function improvement in stroke patients at Prof. Dr. Chairuddin Panusunan Lubis Hospital, University of Sumatera Utara, and affiliated network hospitals

**DOI:** 10.3389/fmed.2026.1769632

**Published:** 2026-04-07

**Authors:** Silvia Fransisca, Bobby Ramses Erguna Sitepu, Delfi

**Affiliations:** Department of Ophthalmology, Faculty of Medicine, University of North Sumatra, Medan, Indonesia

**Keywords:** citicoline, color sensitivity, contrast sensitivity, visual acuity, visual field

## Abstract

**Background:**

Visual disturbances are a common and often debilitating complication following stroke, significantly impairing patients’ functional independence and quality of life. Among various neuroprotective agents, Citicoline (cytidine-5′-diphosphocholine) has demonstrated multifaceted mechanisms of action, including modulation of phospholipid metabolism, reduction of oxidative stress, and enhancement of dopaminergic and cholinergic neurotransmission. These properties suggest potential benefits in promoting visual recovery post-stroke.

**Objectives:**

To analyze the effect of Citicoline administration on the improvement of visual impairment in patients with a history of stroke at Prof. Dr. Chairuddin Panusunan Lubis Hospital, University of Sumatera Utara, and its network hospitals.

**Methods:**

This quasi-experimental study employed a pre- and post-intervention design involving 30 post-stroke patients who received 1,000 mg of oral Citicoline daily for 30 consecutive days. Visual function was evaluated through standard ophthalmological assessments, including visual acuity (logMAR), visual field analysis using the Humphrey Field Analyzer, contrast sensitivity (Pelli-Robson chart), and color sensitivity test (Farnsworth Munsell D-15 test), both before and after treatment.

**Results:**

Post-treatment analysis revealed no statistically significant improvement in visual acuity or visual field parameters (*p* > 0.05). However, contrast sensitivity showed a statistically significant enhancement following Citicoline administration (*p* < 0.05). No significant changes were observed in color vision outcomes (*p* > 0.05).

**Conclusion:**

Oral Citicoline supplementation over a 30-day period appears to exert a beneficial effect specifically on contrast sensitivity in stroke patients with visual dysfunction, while its impact on visual acuity, visual fields, and color perception remains inconclusive. Further research involving combinations with other neuroprotective agents, larger sample sizes, or longer intervention durations is needed to confirm these findings and to explore the potential benefits of Citicoline more comprehensively.

## Introduction

Stroke remains a leading cause of mortality and long-term disability worldwide and can be broadly classified into ischemic and hemorrhagic subtypes, the latter encompassing intracerebral and subarachnoid hemorrhages. Ischemic stroke, characterized by infarction of the brain, spinal cord, or retina, accounts for approximately 71% of all stroke cases globally (1–3). The resultant physical disabilities impart significant morbidity and mortality, substantially compromising patients’ quality of life. Epidemiological data indicate stroke prevalence in Indonesia varies between rural (0.0017%) and urban (0.022%) populations, corresponding to estimated incidences of at least 170 and 2,200 cases per 100,000 individuals, respectively. Nationally, the 2023 Basic Health Research (Riskesdas) reports an overall prevalence of 8.3%, with North Sumatra exhibiting a regional prevalence near 6.6%, and the highest rates observed in the Special Region of Yogyakarta (4, 5).

Post-stroke visual impairment exerts profound functional limitations due to the essential role of vision in daily activities. Clinical manifestations include compromised overall mobility, impaired depth perception, and disrupted stereopsis. These impairments elevate fall risk from collisions on the affected visual field side or from pronounced visual field deficits that markedly reduce visual acuity. Such limitations hinder optimal navigation, safety, and independence, cumulatively diminishing quality of life as part of the post-stroke disability spectrum (6, 7). Furthermore, stroke can adversely affect contrast and color sensitivity, aggravating visual dysfunction and further impairing quality of life (8, 9).

Among neuroprotective agents studied, Citicoline (cytidine-5′-diphosphocholine) has gained considerable attention for systemic neurodegenerative disorders including Alzheimer’s disease, Parkinson’s disease, and cerebral ischemia or infarction. Since its discovery, Citicoline has demonstrated multiple mechanisms of action involving phospholipid homeostasis, redox regulation, mitochondrial dynamics, and modulation of cholinergic and dopaminergic neurotransmission (10, 11). Its neuroprotective potential is attributed to reducing glutamate excitotoxicity and oxidative stress, enhancing neurotrophin levels, promoting neurotransmitter release (e.g., norepinephrine and dopamine), facilitating axonal repair and regeneration, improving mitochondrial function, and modulating insulin signaling pathways. Additionally, Citicoline mitigates free fatty acid accumulation following ischemia, attenuates infarct volume and cerebral edema, and exhibits anti-apoptotic effects through mitochondrial pathways (12, 13).

Citicoline also elevates serotonin levels, augmenting its neuroprotective profile. It lowers glutamate concentrations, limiting excitotoxic damage via N-methyl-D-aspartate (NMDA) receptor inhibition. The restoration of phosphatidylcholine availability further supports neuronal membrane repair and regeneration (10, 14). Moreover, Citicoline inhibits ischemia-induced inflammation by suppressing phospholipase A2 activity, thereby reducing arachidonic acid metabolism, neuroinflammation, and reactive oxygen species production. These combined anti-inflammatory and anti-apoptotic effects reinforce its neuroprotective action (14–16).

Despite the extensive investigation of Citicoline in neurodegenerative contexts, limited studies address its impact on post-stroke visual deficits, particularly regarding contrast and color sensitivity impairments. Previous research by Marino et al. demonstrated Citicoline’s efficacy in enhancing contrast sensitivity in open-angle glaucoma patients, while Parisi et al. highlighted its neuroprotective effects on retinal ganglion cells in retinal degeneration models (17, 18). However, its therapeutic utility for stroke-induced visual dysfunctions remains to be clarified.

Given Citicoline’s multimodal neuroprotection and potential to improve visual acuity, visual fields, contrast, and color perception in stroke, evaluating its clinical benefits in this context is pertinent. This study aims to assess the effects of daily oral Citicoline administration on visual impairments in post-stroke patients.

## Methods

This study employed a quasi-experimental design with a pre- and post-test approach to assess the differences in visual acuity, visual field defects, contrast sensitivity, and color sensitivity before and after administration of 1,000 mg Citicoline in patients with a history of stroke. The research was conducted at the Eye Health Polyclinic, Department of Ophthalmology, Rumah Sakit Prof. Dr. Chairuddin Panusunan Lubis, Universitas Sumatera Utara, along with affiliated network hospitals.

### Study setting and duration

The study was approved by the Ethics Committee of the Faculty of Medicine, Universitas Sumatera Utara (KEK FK USU). Research activities commenced after ethical clearance and spanned from January 2025 until the required sample size was fulfilled.

### Population and sample

The study population consisted of patients diagnosed with visual impairments attributable to stroke at the aforementioned hospitals. A sample of 30 patients fulfilling inclusion criteria was selected. Patients were evaluated for clinical progression of visual impairments—including visual acuity, visual field defects, contrast sensitivity, and color sensitivity—before starting Citicoline treatment and again four weeks after receiving 1,000 mg of oral Citicoline daily.

### Inclusion and exclusion criteria

Inclusion criteria included adults over 18 years of age, diagnosed with ischemic or hemorrhagic stroke, confirmed visual impairments (visual acuity reduction, visual field defect, contrast sensitivity, or color sensitivity disorders) attributable to stroke with ophthalmological verification, medically stable and able to undergo complete ophthalmological examinations per protocol, and willing to undergo treatment as per study protocol. Exclusion criteria included refusal to participate in the entire study protocol, other neurological conditions affecting vision such as Alzheimer’s disease, Parkinson’s disease, or multiple sclerosis, severe head trauma history (Glasgow Coma Scale < 10), visual impairments due to other ocular pathologies such as refractive errors, glaucoma, macular degeneration, or cataract, and known contraindications to Citicoline. Drop-out Criteria included withdrawal of consent during the study, allergic reactions to the investigational drug, loss to follow-up or inability to contact the patient, and non-adherence to medication regimen.

### Sample size calculation

The minimum sample size was estimated using non-inferiority trial formulas, considering a significance level of alpha = 5% (Zα = 1.96), power of 95% (Zβ = 1.64), a meaningful difference in means (*Δ* = 2.9) based on prior literature, and standard deviation (*σ* = 4.0) from visual field defect measurements.

### Data collection instruments

Assessment tools used in the study included Snellen chart for visual acuity, Standard Automated Perimetry (SAP) using Humphrey Field Analyzer for visual field defects, Pelli-Robson chart for contrast sensitivity, Farnsworth-Munsell D-15 test for color sensitivity.

### Ethical considerations

The protocol, including patient consent procedures, was reviewed and approved by the Ethics Committee of FK USU. All participants provided informed consent prior to inclusion.

### Statistical analysis

Data were checked for completeness, coded, and entered into a master dataset in Microsoft Excel 365. Statistical analyses were performed with IBM SPSS. Continuous variables are reported as mean ± standard deviation (SD) and categorical variables as counts (percentages). Repeated measures were compared using repeated-measures ANOVA when normality assumptions were met; otherwise the nonparametric Friedman test was used. Two-sided *p* < 0.05 was considered statistically significant.

## Results

### Demographic characteristics

A total of 30 stroke patients with visual impairments participated in this study. The cohort consisted of 19 males (63.3%) and 11 females (36.7%) with a mean age of 63.5 ± 6.69 years (range: 51–76 years) showed in Table 1.

**Table 1:** Demographic characteristics value.

Characteristic	Value
Gender (Male)	19 (63.3%)
Gender (Female)	11 (36.7%)
Age (Mean ± SD)	63.5 ± 6.69 years

### Visual acuity

Visual acuity was assessed using logMAR values. The median visual acuity before treatment was 1.15 (range 0.8–1.9), and the post-treatment mean was 1.24 ± 0.34 logMAR. Statistical analysis using the Wilcoxon Signed-Rank test revealed no significant difference in visual acuity before and after administration of 1,000 mg oral Citicoline daily (*p* = 0.632).

### Contrast sensitivity

Contrast sensitivity was classified as normal or impaired. Pre-treatment, 50% (*n* = 15) of patients exhibited normal contrast sensitivity, and 50% (*n* = 15) demonstrated impairment. After treatment, 76.7% (*n* = 23) of patients had normal contrast sensitivity, a statistically significant improvement (*p* = 0.008, McNemar test), indicating a positive effect of Citicoline on contrast sensitivity.

### Visual field status

Visual field defects were present in 63.3% (*n* = 19) of patients before treatment, with 36.7% (*n* = 11) having normal visual fields. Post-treatment, abnormal visual field cases slightly increased to 70% (*n* = 21), with normal visual fields decreasing to 30% (*n* = 9). The change was not statistically significant (*p* = 0.500, McNemar test).

## Discussion

This quasi-experimental pre- and post-test study aimed to evaluate the effects of administering 1,000 mg of Citicoline on visual acuity, visual field defects, contrast sensitivity, and color sensitivity in post-stroke patients. The sample majority were male (63.3%, *n* = 19) with a mean age of 63.5 years, consistent with prior reports such as Rowe et al. (19), which found a higher incidence among males (51.5%) with a mean age of 73.3 years in acute stroke populations in the UK.

Visual acuity analysis revealed a median value of 1.15 logMAR prior to intervention and a mean of 1.24 ± 0.34 logMAR after Citicoline administration. Our findings indicate no statistically significant improvement in visual acuity, differing from studies such as Oddone et al. (20), which reported significant gains in visual acuity for patients with non-arteritic anterior ischemic optic neuropathy (NAION) treated with oral Citicoline (1,600 mg/day) in cyclic regimens. Parisi et al. (21) also observed improvements in visual acuity and neurophysiological markers (PERG and VEP) after 6 months of oral Citicoline therapy in NAION patients. The divergent results may reflect differences in dosage, treatment duration, pathology, and the presence of wash-out periods.

Contrast sensitivity improved significantly following Citicoline treatment, suggesting a beneficial effect on this visual parameter in stroke patients. This aligns with Marino et al. (18), who documented enhanced contrast sensitivity in glaucoma patients receiving daily supplementation of Citicoline combined with homotaurine and vitamin E, measured by SPARCS scores. The mechanism remains unclear, though Citicoline’s dopaminergic activity and retinal effects, including increased retinal dopamine levels observed in animal models, may play a role. Additionally, citicoline has been reported to upregulate SIRT1 (sirtuin-1), a NAD + -dependent deacetylase implicated in mitochondrial biogenesis, anti-apoptotic signalling and suppression of neuroinflammation — mechanisms that together promote neuronal survival in ischemic stroke and neurodegenerative disease. Experimental studies demonstrate citicoline-associated increases in SIRT1 concomitant with neuroprotection (22), and recent reviews summarize citicoline’s modulation of SIRT1-related pathways as a plausible mediator of its pleiotropic effects (23, 24).

Visual field defects showed no significant change after treatment, consistent with Anton et al. (25), who found no improvement in visual fields among glaucoma patients during Citicoline therapy. Contrarily, Parisi et al. (21) reported visual field improvements in NAION patients. The cognitive-enhancing properties of Citicoline, such as increased alertness, might explain slight visual field changes observed in glaucoma patients as reported by Ottobelli et al. (26).

Color sensitivity was unaffected by Citicoline treatment, with the proportion of patients exhibiting deuteranopia remaining stable at 10%. Color vision deficiency (CVD) is primarily a congenital disorder characterized by impairments in red-green differentiation due to defective cone photopigments (27, 28). Citicoline’s neuroprotective action on retinal ganglion cells does not extend to direct modulation of cone photopigments, aligning with our findings.

Finally, our treatment and follow-up period was short (30 days). Several clinical trials and mechanistic reports indicate that citicoline’s pleiotropic neuroprotective actions (membrane repair, mitochondrial support, anti-inflammatory and anti-apoptotic pathways) often become measurable only after sustained administration of several months; randomized studies using citicoline interventions for visual/neuro-functional outcomes commonly used at least three months of treatment to show the best results (25). Therefore, the short duration of our study may have underestimated the magnitude and timeline of citicoline’s benefits.

Limitations of the study include the absence of stroke duration data, lack of wash-out periods for neuroprotective agents, and a relatively small sample size. Future investigations with larger cohorts and rigorous controls are warranted to fully elucidate Citicoline’s role in visual rehabilitation post-stroke.

## Conclusion

This study investigated the effects of oral Citicoline supplementation over a 30-day period in stroke patients with visual dysfunction. The majority of subjects were male (63.3%) with a mean age of 63.5 years. Before intervention, median visual acuity was 1.15 logMAR (range 0.8 to 1.9), which slightly changed to a mean of 1.24 ± 0.34 logMAR after Citicoline treatment. The number of patients with abnormal visual fields increased to 70% post-intervention, while those with normal visual fields decreased to 30%. In contrast, the proportion of patients with normal contrast sensitivity improved significantly, rising to 76.7% following treatment, whereas those with impaired contrast sensitivity reduced to 23.3%. Color perception, specifically deuteranopia, remained unchanged, affecting 10% of patients before and after intervention.

Statistical analysis demonstrated that Citicoline administration had no significant effect on visual acuity (*p* = 0.632), visual fields (*p* = 0.500), or color sensitivity (*p* = 1.000). However, Citicoline significantly improved contrast sensitivity (*p* = 0.008) in stroke patients with visual impairment. These findings suggest a specific beneficial role of Citicoline in enhancing contrast sensitivity, while its influence on other visual parameters is inconclusive.

The limited effects observed on visual acuity, visual fields, and color sensitivity may reflect the short, 30-day treatment period: citicoline’s pleiotropic neurorestorative mechanisms (membrane phospholipid synthesis, modulation of neurotransmitters, and promotion of neuroplasticity) and the structural and functional recovery they support typically require sustained administration over weeks to months to accumulate and become clinically measurable.

Given the limited sample size and short intervention period, further research with larger cohorts, extended treatment duration, and potential combination with other neuroprotective agents is warranted to comprehensively evaluate the neurovisual benefits of Citicoline in post-stroke rehabilitation.

## Data Availability

The raw data supporting the conclusions of this article will be made available by the authors, without undue reservation.
